# The likelihood of persistent arthritis increases with the level of anti-citrullinated peptide antibody and immunoglobulin M rheumatoid factor: a longitudinal study of 376 patients with very early undifferentiated arthritis

**DOI:** 10.1186/ar2995

**Published:** 2010-05-05

**Authors:** Maria D Mjaavatten, Désirée van der Heijde, Till Uhlig, Anne J Haugen, Halvor Nygaard, Göran Sidenvall, Knut Helgetveit, Tore K Kvien

**Affiliations:** 1 Department of Rheumatology, Diakonhjemmet Hospital, P.O. Box 23 Vinderen, 0319 Oslo, Norway; 2 Department of Rheumatology, Leiden University Medical Center, Albinusdreef 2, Leiden, P.O. Box 9600, 2300RC, The Netherlands; 3 Department of Rheumatology, Østfold Hospital Trust, 1603 Fredrikstad, Norway; 4 Lillehammer Hospital for Rheumatic Diseases, Margrethe Grundtvigs vei 6, 2609 Lillehammer, Norway; 5 Department of Rheumatology, Innlandet Hospital, 2226 Kongsvinger, Norway; 6 Martina Hansen Hospital, P.O. Box 23, 1306 Bærum Postal Terminal, Norway

## Abstract

**Introduction:**

We wanted to assess the importance of the levels of anti-citrullinated peptide antibody (anti-CCP) and immunoglobulin M (IgM) rheumatoid factor (RF) in predicting development of persistent arthritis from undifferentiated arthritis (UA), and to investigate whether there is an added predictive value for persistent arthritis in testing for both anti-CCP and IgM RF.

**Methods:**

Patients with UA (exclusion of definite non-rheumatoid arthritis (RA) diagnoses) included in the Norwegian very early arthritis clinic were assessed for development of persistent arthritic disease. The effect of antibody level on the likelihood of persistent arthritis was investigated, and the sensitivity and specificity for persistent arthritis for anti-CCP and IgM RF, separately and combined, was determined.

**Results:**

A total of 376 UA patients were included (median arthritis duration 32 days). 59 (15.7%) patients were IgM RF positive, and 62 (16.5%) anti-CCP positive. One hundred, seventy-four (46.3%) had persistent disease after one year. Overlap of anti-CCP and IgM RF positivity was 58%. Sensitivity/specificity for persistent arthritis was 28/95% for IgM RF alone, 30/95% for anti-CCP alone, and 37/92% for positivity of both anti-CCP and IgM RF. The likelihood for persistent disease increased with increasing levels of both anti-CCP and IgM RF.

**Conclusions:**

The likelihood of developing persistent arthritis in UA patients increases with the level of anti-CCP and IgM RF. Testing both anti-CCP and IgM RF has added predictive value in UA patients. This study suggests that antibody level should be taken into account when making risk assessments in patients with UA.

## Introduction

Rheumatoid factor (RF) has traditionally been regarded as the main serologic marker in inflammatory arthritis [1,2]. In recent years anti-citrullinated protein antibodies (ACPA), most commonly measured by assays for antibodies against cyclic citrullinated peptide (anti-CCP), have also been identified as important predictors both for diagnosis and prognosis in rheumatoid arthritis (RA) [3,4]. RF has similar sensitivity as anti-CCP in RA diagnosis but lower specificity [3,5-8], and RF and anti-CCP are both independent predictors of erosive progression [9].

The paradigm of a *window of opportunity *in the treatment of inflammatory arthritis has raised awareness of seeing patients at the earliest possible stage of disease. The 1987 American Rheumatism Association (ARA) classification criteria for RA [1] do not perform well in early disease [10] and early arthritis is often undifferentiated and may develop into RA [11,12]. A few studies have identified anti-CCP or RF as predictors of persistent arthritis (as opposed to remission of disease) [13-17].

Several questions remain unanswered regarding the predictive role of anti-CCP and RF in patients with early undifferentiated arthritis. What is the optimal cut-off level for defining a positive antibody status? Is a *high positive *level of anti-CCP or RF more predictive of an unfavourable outcome than a *low positive *level? What is the added value (if any) of testing for both markers? Regarding the optimal cut-off of anti-CCP level, a recent study on pre-RA sera [18] suggested that lowering thresholds below that of the manufacturer's recommended cut-off level gave more sensitive prediction of future RA development. Increased levels of ACPA are associated with worse radiographic progression and higher disease activity in RA [9,19,20], whereas no such relationship was found in a recent study of prognosis in early arthritis patients [21]. No studies have assessed the predictive value of the levels of anti-CCP and RF in patients with early undifferentiated arthritis.

The objectives of this study were 1) to investigate whether there is an added predictive value for persistent arthritis in testing for both anti-CCP and IgM RF and 2) to assess the predictive performance for persistent arthritis of the level of anti-CCP and RF in patients with arthritis duration <16 weeks.

## Materials and methods

### Early arthritis clinic

The Norwegian Very Early Arthritis Clinic (NOR-VEAC) study was started in 2004 as a multicenter observational study in the South-Eastern part of Norway. The five participating hospitals serve a region with approximately 1.7 million inhabitants. The cohort includes patients (age 18 to 75) presenting with at least one clinically swollen joint of ≤ 16 weeks duration, and patients are followed longitudinally for two years. One year outcome was used in the present study.

Joint swelling due to trauma, osteoarthritis, crystal arthropathies, and septic arthritis are exclusion diagnoses; if any of these diagnoses are made during follow-up, patients are excluded from further follow-up. The details of the data collection have been described elsewhere [22], and are summarised in Additional file 1. Imaging procedures were not a part of the data collection for the patients included in this analysis. The study was approved by the regional Ethics Board and the Data Inspectorate, and patients gave an informed consent.

### Laboratory markers

Erythrocyte sedimentation rate (ESR) and C-reactive protein levels were determined locally at the participating centres. Serum was frozen at baseline and stored at -70°C and used to analyse anti-citrullinated protein antibodies (ACPA) (anti-CCP2, INOVA Diagnostics, Inc.^® ^San Diego, CA, USA) and IgM rheumatoid factor (RF) (in-house enzyme-linked immunosorbent assay) [9]. Analyses of the serologic markers were performed in one batch. Anti-CCP levels were reported in units from 2 to 250. Any level greater than 250 was reported as >250 and analysed as 251, and levels less than 2 were reported as <2 and analysed as 1. Similarly, IgM RF levels were reported in units from 2 to 300. Any level greater than 300 was reported as >300 and analysed as 301, and levels less than 2 were reported as <2 and analysed as 1. The cut-off levels recommended by the central laboratory for positivity of the serologic markers are: Anti-CCP2 ≥ 25 units/ml, IgM RF ≥ 25 units/ml [9].

### Patient selection and outcome determination

Patients included before 1 June 2007, who had baseline information about IgM RF and anti-CCP, were eligible for the current analysis. Patients who were judged by the treating rheumatologist to have a definite diagnosis other than RA were excluded from the present analysis, leaving only patients with very early undifferentiated arthritis in the study. Persistent arthritis (the outcome) was defined as presence of joint swelling at one year. For patients lost to follow-up before one year, the last registered follow-up visit was used in a *last observation carried forward *(LOCF) manner with regard to joint swelling, provided that at least one follow-up visit was recorded. Patients prescribed with disease-modifying anti-rheumatic drug (DMARD) therapy within one year of presentation were also included in the persistent arthritis group. To determine whether it was correct to include these DMARD-starting patients in the persistent arthritis group, baseline characteristics were compared between patients with persistent synovitis (with or without concomitant DMARD prescription) and patients in which DMARDs were prescribed without coexisting persistent arthritis.

### Statistical analysis

Means and standard deviations were calculated for continuous variables following a Gaussian distribution, otherwise median values and inter quartile ranges (25^th ^to 75^th ^percentiles) were calculated. Independent samples T-tests/Mann-Whitney-U tests were used for group comparisons where appropriate. Frequencies were calculated for categorical variables and compared using Chi-square tests.

The relationship between anti-CCP as a continuous/dichotomised variable and the outcome (persistent arthritis) was investigated through univariate and subsequently multivariate logistic regression analyses. The multivariate analyses were adjusted for factors previously found to affect the outcome (age, sex, small joint arthritis, functional status according to Health Assessment Questionnaire (HAQ) score, 28-tender joint count, C-reactive protein, morning stiffness >1 hour) [13,16,23]. IgM RF and anti-CCP were assessed as continuous variables with receiver operating characteristic (ROC) curve analysis. ROC curve analysis was also used to investigate whether alternative cut-offs had more optimal combined sensitivity/specificity than the recommended cut-off of 25 units/ml. Likelihood ratios and their 95% confidence intervals were calculated for these alternative cut-offs.

To assess the effect of increasing levels of anti-CCP and IgM RF on the likelihood of developing persistent disease, anti-CCP and IgM RF levels were divided into ordinal categories (based on the recommended cut-off as well as with tertiles/median of antibody positive patients for anti-CCP and IgM RF, respectively). This resulted in two new, categorized variables (anti-CCP: ≤ 25, >25 to 100, >100 to 250, >250 units/ml; IgM RF: ≤ 25, >25 to 75, >75 units/ml), and these variables were entered into separate univariate logistic regression models. Likelihood ratios for each of the categories compared to the reference category (≤ 25 units/ml) were calculated. The prediction models were also tested in each subgroup of patients with persistent disease (persistent synovitis or DMARD prescription alone) as a sensitivity analysis.

## Results

Baseline information about anti-CCP and IgM RF was available in 483/572 (84%) patients enrolled in the study. Patients with and without available sera were similar regarding baseline characteristics and outcome (data not shown). Eighty-nine patients were excluded from the analysis because they were diagnosed with a specific rheumatological non-RA condition at the initial assessment (Löfgren's syndrome/sarcoidosis-associated arthritis 39, psoriatic arthritis according to the Moll & Wright criteria for psoriatic arthritis (PsA) [24] 38, gout 10, ulcerative colitis-arthritis 1, polymyalgia rheumatica 1). Further, 18 patients were excluded from analysis because they had no follow-up data recorded, leaving 376 patients with very early undifferentiated inflammatory arthritis for the current analysis.

Baseline characteristics of the 376 patients are presented in Table 1. The baseline data were complete except for some missing data for morning stiffness. Median (interquartile range, IQR) anti-CCP level in the 62 (16.5%) patients who were anti-CCP2 positive (cut-off 25 units/ml) was 205 (76 to 251) units/ml. Fifty-nine (15.7%) patients were IgM RF positive (>25 units/ml), with median (IQR) IgM RF level 69 (44 to 125) units/ml). Twenty-one patients were solely anti-CCP positive and 18 solely RF positive, while 41 patients were positive for both serologic markers. One hundred seventy-four (46.3%) patients had persistent disease after one year (persistent joint swelling 65, DMARD prescribed 52, both 57). In 32 patients the outcome was determined by the LOCF strategy due to missing data (persistent arthritis 9, remission 23). The DMARDs prescribed were distributed as follows: methotrexate 72 (66.1%) patients, sulphasalazine 21 (19.3%), hydroxychloroquine 4 (3.7%), biologics (+ methotrexate) 5 (1.3%), combinations of traditional DMARDs 5 (1.3%), other 2 (0.5%) patients. Patients with and without persistent disease at one year differed significantly with respect to several baseline characteristics (Table 1).

**Table 1:** Baseline characteristics of the patients with comparison between patients with/without persistent arthritis at one year

	All patients (n = 376)	Persistent disease (N = 174)		Self-limiting disease (N = 202)	P*
				
		Persistent synovitis (with or without DMARD) (N = 122)	DMARD prescribed **(without persistent synovitis)**^$^ (N = 52)		
Female gender^a^	218 (58.0)	78 (63.9)	32 (61.5)	108 (53.5)	0.06
Age, years^b^	46.3 (14.8)	49.0 (14.9)	45.6 (17.1)	44.9 (14.0)	0.04
Arthritis duration, days^c^	32 (10 to 64)	50 (21 to 69)	59 (27 to 76)	21 (7 to 49)	<0.001
SJC (0 to 68)^c^	2 (1 to 5)	4 (1 to 9)	4 (2 to 8)	1 (1 to 3)	<0.001
TJC (0 to 28)^c^	1 (1 to 3)	2 (1 to 6)	2 (1 to 5)	1(0 to 2)	<0.001
ESR, mm/h^c^	24 (11 to 46)	27 (10 to 50)	32 (17 to 51)	19 (10 to 38)	0.005
CRP, mg/l^c^	15.0 (5.0 to 36.4)	15 (5 to 35)	18 (9 to 45)	14.0 (3.5 to 36.2)	0.16
IgM RF level, units/ml^c^	4 (1 to 10)	6 (2 to 40)	6 (2 to 30)	3 (1 to 7)	<0.001
IgM RF positive^a^	59 (15.7)	36 (29.5)	13 (25.0)	10 (5.0)	<0.001
Anti-CCP2 level, units/ml^c^	3 (2 to 6)	4 (2 to 83)	4 (2 to 25)	2 (2 to 4)	<0.001
Anti-CCP2 positive^a^	62 (16.5)	39 (32.0)	13 (25.0)	10 (5.0)	<0.001
Assessor's global VAS, mm^b^	35.8 (20.7)	39.5 (21.8)	44.7 (23.0)	31.3 (18.2)	<0.001
Patient's global VAS, mm^b^	53.8 (24.0)	57.4 (21.9)	59.5 (26.1)	50.2 (24.1)	0.001
Small joint arthritis^a^	173 (46.0)	73 (59.8)	33 (63.5)	67 (33.2)	<0.001
Morning stiffness>1 hour^a^	195 (51.9)^§^	70 (57.9)	31 (60.8)	101 (58.0)	0.04
DAS28^b^	4.05 (1.32)	4.47 (1.35)	4.61 (1.36)	3.65 (1.15)	<0.001
HAQ (0-3)^b^	0.89 (0.67)	0.98 (0.64)	1.07 (0.80)	0.78 (0.63)	0.001
Monoarthritis^a^	154 (41.0)	37 (30.3)	12 (23.1)	105 (52.0)	<0.001
-of the knee^a^	80 (51.9)	20 (54.1)	6 (50.0)	54 (51.4)	0.85
Fulfilment of 1987 ACR criteria^a†^	70 (18.6)	37 (30.3)	16 (30.8)	17 (8.4)	<0.001

ACR: American College of Rheumatology; anti-CCP2: 2nd generation anti-citrullinated peptide antibody; CRP: C-reactive protein; DAS: Disease Activity Score; ESR: erythrocyte sedimentation rate; HAQ: Health Assessment Questionnaire; RF: rheumatoid factor; SJC: swollen joint count; TJC: tender joint count; VAS: visual analogue scale; small joint arthritis, joint swelling in PIP-, MCP- or MTP joint(s).

^$^No statistical differences between the persistent arthritis group and DMARD group for any of the baseline variables were found.

*Comparing persistent disease group with self-limiting disease group.^a^Count (%).^b^Mean (standard deviation).^c^Median (inter-quartile range).^§^N = 369.^†^Modified from the 1987 ARA criteria according to available data (excluding criteria on erosive disease and duration of symptoms).

### Associations between persistent disease and the serologic markers

Logistic regression analyses showed significant univariate associations with persistent disease for anti-CCP and IgM RF which were maintained in the multivariate analysis (Table 2). The odds ratios for persistence were almost doubled when both serologic markers were present.

**Table 2:** Logistic regression analysis exploring the relationship between anti-CCP/IgM RF and persistent arthritic disease

	Univariate			Multivariate*		
	
	estimate	OR (95% CI)	*P*	estimate	OR (95% CI)	*P*
Anti-CCP2 (units/ml)	0.013	1.013 (1.008 to 1.018)	<0.001	0.008	1.008 (1.003 to 1.013)	0.002
Anti-CCP2+ (>25 units/ml)	2.102	8.184 (4.008 to 16.709)	<0.001	1.272	3.567 (1.572 to 8.094)	0.002
IgM RF (units/ml)	0.022	1.022 (1.012 to 1.033)	<0.001	0.009	1.009 (1.001 to 1.017)	0.026
IgM RF+ (>25 units/ml)	2.018	7.526 (3.677 to 15.407)	<0.001	0.999	2.716 (1.189 to 6.202)	0.018
IgM RF+ and anti-CCP+	2.593	13.369 (4.658 to 38.369)	<0.001	2.073	7.948 (2.659 to 23.752)	<0.001

*Adjusted for age, sex, small joint arthritis (swelling of MTP-, PIP- and/or MCP joints), Health Assessment Questionnaire, 28-tender joint count, C-reactive protein (mg/l), morning stiffness >1 hour, as well as IgM rheumatoid factor (for the analysis with anti-CCP) or anti-CCP (for the analysis with IgM RF), respectively.

The distribution of anti-CCP- and RF positive patients with and without persistent disease is shown in Table 3. Only 58% (37/64) of antibody positive patients with persistent arthritis were positive for both RF and anti-CCP. Testing for only anti-CCP would identify 52 of the 174 patients (sensitivity 30%) with persistent arthritis. Specificity was 95.0%. Testing for only IgM RF would identify slightly fewer patients, 49/174 (sensitivity 28%), with equal specificity as for anti-CCP alone (95.0%). Testing for both markers would identify 64/174 patients (sensitivity 37%). The number of false-positives for anti-CCP and RF combined was 16 patients, yielding a specificity of 92.1% for the *and/or *combination. A *serial *measuring strategy starting with measuring IgM RF would not identify 21/80 (26.3%) autoantibody positive patients, and 15/174 (8.6%) of patients with persistent arthritis would be missed.

**Table 3:** Distribution of anti-CCP- and IgM RF positive patients with/without persistent arthritis

	Persistent arthritis	Self-limiting disease	Total
Anti-CCP positive only	15	6	21
Anti-CCP and IgM RF positive	37	4	41
IgM RF positive only	12	6	18
No antibody positivity	110	186	296
Total	174	202	376

### ROC curve analysis and alternative cut offs

**Figure 1:**
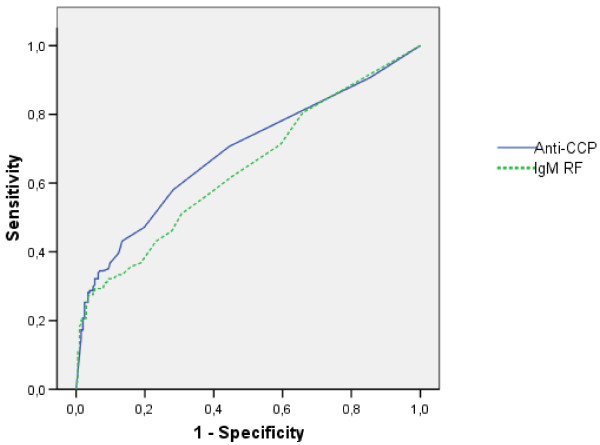
**Receiver operating characteristic curve analysis**. Receiver operating characteristic (ROC) curve analysis investigating the discriminatory capacity for persistent arthritis at one year of baseline antibody levels. Area under the curve (AUC) (95% confidence interval): Anti-CCP: 0.687 (0.633 to 0.742), IgM RF: 0.641 (0.585 to 0.697).

The ROC curve analysis showed moderate discriminatory capacity of anti-CCP (Figure 1), with an area under the curve (AUC) of 0.69. The combination of slightly increased accuracy and positive likelihood ratios remaining above 4 suggested some gain in informative value of lowering the threshold down to about 50% of the recommended cut-off for anti-CCP (Table 4). The ROC curve analysis for IgM RF showed slightly less discriminatory capacity than anti-CCP, with an AUC of 0.64 (Figure 1). Lowering the threshold for defining a positive status for RF resulted in a rather large loss of specificity and positive likelihood ratio (LR) with little gain in sensitivity or negative LR (Table 4).

**Table 4:** Sensitivity, specificity, and likelihood ratios for persistent arthritis for different minimum cut-off levels for anti-CCP2 and IgM RF

Cut-off anti-CCP2 (units/ml)	Sensitivity	Specificity	Accuracy	LR+ (95% CI)	LR- (95% CI)
2	0.71	0.55	0.63	1.59 (1.32 to 1.91)	0.53 (0.41 to 0.69)
3	0.58	0.72	0.65	2.06 (1.60 to 2.65)	0.58 (0.48 to 0.71)
4	0.47	0.80	0.65	2.38 (1.73 to 3.27)	0.66 (0.56 to 0.77)
5	0.43	0.87	0.67	3.23 (2.18 to 4.77)	0.66 (0.57 to 0.77)
10	0.35	0.93	0.66	4.98 (2.88 to 8.58)	0.71 (0.69 to 1.79)
15	0.32	0.95	0.66	5.91 (3.20 to 10.92)	0.72 (0.64 to 0.80)
20	0.31	0.95	0.65	5.59 (3.02 to 10.37)	0.74 (0.66 to 0.82)
25	0.30	0.95	0.64	6.04 (3.17 to 11.51)	0.74 (0.67 to 0.82)
Cut-off IgM RF (units/ml)	Sensitivity	Specificity	Accuracy	LR+	LR-

2	0.71	0.41	0.55	1.20 (1.04 to 1.39)	0.71 (0.53 to 0.94)
3	0.62	0.54	0.58	1.34 (1.13 to 1.65)	0.70 (0.55 to 0.88)
5	0.51	0.69	0.61	1.67 (1.30 to 2.15)	0.71 (0.59 to 0.84)
7	0.43	0.77	0.61	1.85 (1.37 to 2.51)	0.74 (0.64 to 0.86)
10	0.33	0.87	0.62	2.49 (1.66 to 3.76)	0.77 (0.68 to 0.87)
15	0.31	0.92	0.64	3.69 (2.22 to 6.12)	0.75 (0.68 to 0.84)
20	0.29	0.95	0.64	5.28 (2.84 to 9.81)	0.75 (0.68 to 0.83)
25	0.28	0.95	0.64	5.69 (2.97 to 10.89)	0.76 (0.69 to 0.83)

CI: confidence interval; LR+: positive likelihood ratio; LR-: negative likelihood ratio.

### Effect of increasing antibody levels

When patients were divided into categories based on anti-CCP level (as described in the Methods section) the numbers of patients in each category were as follows: ≤ 25 units/ml (n = 314), >25 to 100 units/ml (n = 19), >100 to 250 units/ml (n = 14), >250 units/ml (n = 29). The likelihood for having persistent arthritic disease increased with increasing levels of anti-CCP (Table 5), suggesting that the level of ACPA can be informative in prediction of persistent arthritis.

**Table 5:** Univariate logistic regression for persistent arthritis with anti-CCP/IgM RF as categorical (grouped) variables according to level

Anti-CCP (units/ml)	OR (95% C.I.)	*P*	LR+	LR-	Persistent arthritis patients/patients in category n/N
≤ 25	1.0		ref	ref	122/314 (38.9%)
>25 to 100	4.4 (1.6 to 12.5)	0.005	4.1 (1.5 to 11.0)	0.9 (0.9 to 1.0)	14/19 (73.7%)
>100 to 250	9.4 (2.1 to 42.9)	0.004	8.7 (2.0 to 38.2)	0.9 (0.8 to 1.0)	12/14 (85.7%)
>250	13.6 (4.0 to 46.0)	<0.001	11.4 (3.5 to 37.0)	0.8 (0.8 to 0.9)	26/29 (89.7%)
IgM RF (units/ml)	OR (95% C.I.)	*P*	LR+	LR-	

≤ 25	1.0		ref	ref	125/317 (39.4%)
>25 to 75	4.6 (2.0 to 10.6)	<0.001	4.0 (1.9 to 8.7)	0.9 (0.8 to 0.9)	24/32 (75.0%)
>75	19.2 (4.5 to 82.5)	<0.001	16.2 (3.9 to 67.2)	0.8 (0.8 to 0.9)	25/27 (92.6%)

CI: confidence interval; LR+: positive likelihood ratio; LR-: negative likelihood ratio; OR, odds ratio

Similarly, the numbers of patients in each of the categories for IgM RF level were: ≤ 25 units/ml (n = 317), >25 to 75 units/ml (n = 32), >75 units/ml (n = 27). As for anti-CCP, increasing levels of IgM RF were associated with higher positive likelihood of developing persistent arthritis. The negative likelihood ratios for all cut-offs were rather high but consistent with increasing levels. Sensitivity analyses with DMARD start alone or persistent synovitis alone as outcome did not change these results.

## Discussion

This study is the first to show that the likelihood of persistent arthritis increases with the levels of IgM RF and anti-CCP. We also demonstrate an added predictive value in testing for both biomarkers in patients with very early undifferentiated arthritis. Several studies have assessed anti-CCP and/or RF as predictors of persistent arthritic disease in patients with undifferentiated arthritis [7,13,15-17] but none of these investigated the influence of antibody level on diagnostic outcome. The association between levels of these serologic markers and outcome also supports the new American College of Rheumatology (ACR)/European League Against Rheumatism (EULAR) classification criteria for RA which provide different points in the classification system according to level of anti-CCP or RF. (Aletaha D, *et al*. The 2010 American College of Rheumatology/European League Against Rheumatism Classification Criteria for Rheumatoid Arthritis. *Arthritis Rheum/Ann Rheum Dis *2010, submitted.)

When working with prediction models, the challenge of circularity arises when the predictive factor is a part of diagnostic or classification criteria. Presence of the factor of interest can bias the physician to label or diagnose a patient (for example, rheumatoid factor or anti-CCP and RA) [13,25]. This challenge can be overcome by using alternative definitions of the outcome, like persistent joint swelling or erosive disease, which are examples of more objective outcomes in early arthritis patients. Their determination relies solely on the presence of physical features, and therefore is less likely to be biased. Moreover, 17 patients who fulfilled the 1987 ARA criteria for RA at baseline turned out to have self-limiting disease after one year (Table 1), which is in line with previous early arthritis studies demonstrating the low specificity of these criteria [10,26-28].

As evidence of the superior specificity and similar sensitivity of anti-CCP as compared to RF as a predictor of an adverse outcome in inflammatory arthritis has increased [3,5-7], the question of whether one should stop testing for RF has been raised [29]. The overlap between RF and anti-CCP positivity may be greater in established RA (93% in a study of 784 RA patients, mean disease duration 18 years) [30], than in early arthritis (50 to 80% in different studies) [5,7,31]. In the present study only 58% (37/64) of antibody positive patients with persistent arthritis were positive for both RF and anti-CCP. Testing for both markers increased sensitivity for persistent arthritis from about 30% to 37%. Our results support that each of these antibodies should be evaluated when making risk assessments in early arthritis, especially if the one first tested is negative.

We also investigated the benefits and drawbacks of lowering the threshold for defining a positive antibody status. Although lowering the threshold resulted in a rather steep loss of specificity for IgM RF, we found some, but limited, added predictive value of lowering the threshold for anti-CCP positivity to 10 units/ml. This is in line with the findings in a recent study [18]. We believe that the clinician should be aware of the potential value also of low levels of anti-CCP when evaluating early arthritis patients, but we cannot recommend a change in the current threshold for positivity based on our findings.

Is there a *dose-response relationship *between antibody level and likelihood of developing persistent arthritis? Kudo-Tanaka and colleagues reported a higher mean level of anti-CCP2 (Axis-Shield Diagnostics Ltd., Dundee, Scotland, cut-off 4.6 U/ml for positive status) in patients developing RA (167 U/ml) than in patients developing other arthropathies or persistent UA (55 U/ml) in a study of 146 anti-CCP positive UA patients [32]. A Dutch study of patients with arthralgia demonstrated higher median levels of ACPA and IgM RF in patients developing arthritis than in patients who did not [33]. Our findings support that patients with very high levels of anti-CCP or rheumatoid factor are more prone to enter a serious disease course than individuals with low positive levels. This study is the first to demonstrate the effect of levels of anti-CCP and IgM RF on the likelihood of persistent disease, although the numbers of patients with a positive antibody status is moderate and the confidence intervals for the point estimates are wide. A dose-response relationship could support the theory of a pathophysiologic role for anti-CCP antibodies in the development of RA and persistent arthritic diseases, but further studies are needed to shed more light on this complex issue. Importantly, our study supports that presence of high antibody levels give more points than low levels in the new ACR/EULAR classification criteria for RA.

We have shown in this and a previous report [23] that anti-CCP is the most important predictor of an adverse outcome in early arthritis. However, as most patients who developed persistent disease were antibody negative, it is important to bear in mind that early referral and assessment is needed in all patients with significant inflammatory arthritis.

## Conclusions

This study of persistent arthritis in patients with arthritis of less than 16 weeks' duration demonstrates that high positive levels of IgM RF and anti-CCP, as compared to low positive levels increase the likelihood of developing chronic arthritic disease, and also suggests an added value of testing for both antibodies in early disease. We conclude that antibody level should be taken into account when making risk assessments in patients with early undifferentiated arthritis.

## Abbreviations

ACPA: anti-citrullinated protein antibodies; ACR: American College of Rheumatology; anti-CCP: anti-citrullinated peptide antibody; anti-CCP2: 2nd generation anti-citrullinated peptide antibody; ARA: American Rheumatism Association; AUC: area under the curve; CRP: C-reactive protein; DAS: Disease Activity Score; DMARD: disease-modifying anti-rheumatic drug; ESR: erythrocyte sedimentation rate; HAQ: Health Assessment Questionnaire; IgM: immunoglobuline M; IQR: inter-quartile range; LOCF: last observation carried forward; LR: likelihood ratio; NOR-VEAC: Norwegian Very Early Arthritis Clinic; PsA: psoriatic arthritis; RA: rheumatoid arthritis; RF: rheumatoid factor; ROC: receiver operating characteristic; SJC: swollen joint count; TJC: tender joint count; UA: undifferentiated arthritis; VAS: visual analogue scale

## Competing interests

The authors declare that they have no competing interests.

## Authors' contributions

MDM performed the statistical analyses and drafted the manuscript, as well as participated in the study design. DvdH contributed to the analysis and interpretation of data and helped draft the manuscript. TU helped to draft the manuscript and participated in the data collection. AJH, HN and KH participated in the study design and data collection. GS participated in the data collection. TKK was the main designer of the study and helped draft the manuscript. All authors read and approved the final manuscript.

## Supplementary Material

Additional file 1 **Summary of data collection NOR-VEAC**. MS Word file containing a summary of data collection NOR-VEAC. Click here for file
