# Supplementary material for: The likelihood of persistent arthritis increases with the level of anti-citrullinated peptide antibody and immunoglobulin M rheumatoid factor: a longitudinal study of 376 patients with very early undifferentiated arthritis
Source: Arthritis Res Ther. 2010 May 5;12(3):R76. doi: 10.1186/ar2995 (PMC2911852; doi:10.1186/ar2995)
Supplement: Additional file 1 — Summary of data collection NOR-VEAC. MS Word file containing a summary of data collection NOR-VEAC. [file ar2995-S1.DOC]

**Additional file 1.** Summary of data collection NOR-VEAC

The initial purpose of the Norwegian Very Early Arthritis Clinic (NOR-VEAC) study was to investigate patient characteristics and disease outcomes after two years in patients (age 18-75) presenting with at least one clinically swollen joint of ≤16 weeks duration. Primary care physicians in the area received a letter with a request and an opportunity to refer such patients. Referral could be performed by telephone or letter, and patients were guaranteed a consultation with a rheumatologist within 14 days. In order to increase awareness of inflammatory arthritis, the general practitioners were invited to attend evening courses which focused on the importance of early diagnosis and practical training in joint examinations.

All referred patients were managed and followed according to clinical judgement. The patients were considered with regard to the inclusion criteria (arthritis of ≤16 week's duration) and exclusion criteria (joint swelling due to trauma, osteoarthritis and septic arthritis) of the research part of the project, and eligible patients were then asked to sign an informed consent. Data from consenting patients were entered into a research database. Data collection was performed by rheumatologists and designated study nurses in the different centres. Registration included age, sex, duration of symptoms, co-morbidities, extra-articular symptoms, level of education, occupational status, smoking and coffee drinking habits, height and weight. 68-swollen joint counts and 28-tender joint counts were performed by a rheumatologist or by experienced study nurses. Patient-reported outcomes included joint pain, fatigue, and global health status on visual analogue scales (VAS), the Norwegian versions of the Health Assessment Questionnaire (HAQ), Short Form Health Survey (SF-36), and Rheumatoid Arthritis Disease Activity Index (RADAI). The assessor reported patient global health on a VAS scale, as well as DMARD treatment.
